# Dysregulation of AMPA receptor subunit expression in sporadic ALS post‐mortem brain

**DOI:** 10.1002/path.5351

**Published:** 2019-11-23

**Authors:** Jenna M Gregory, Matthew R Livesey, Karina McDade, Bhuvaneish T Selvaraj, Samantha K Barton, Siddharthan Chandran, Colin Smith

**Affiliations:** ^1^ Centre for Clinical Brain Sciences University of Edinburgh Edinburgh UK; ^2^ Euan MacDonald Centre for Motor Neurone Disease Research University of Edinburgh Edinburgh UK; ^3^ Centre for Discovery Brain Sciences University of Edinburgh Edinburgh UK

**Keywords:** sporadic, ALS, SOD1, C9orf72, AMPAR, neuron, post‐mortem, RNA, BaseScope

## Abstract

Amyotrophic lateral sclerosis (ALS) is characterised by progressive motor neuron degeneration. Although there are over 40 genes associated with causal monogenetic mutations, the majority of ALS patients are not genetically determined. Causal ALS mutations are being increasingly mechanistically studied, though how these mechanisms converge and diverge between the multiple known familial causes of ALS (fALS) and sporadic forms of ALS (sALS) and furthermore between different neuron types, is poorly understood. One common pathway that is implicated in selective motor neuron death is enhanced α‐amino‐3‐hydroxyl‐5‐methyl‐4‐isoxazole‐propionate (AMPAR)‐mediated excitoxicity. Specifically, human *in vitro* and pathological evidence has linked the *C9orf72* repeat expansion mutation to a relative increase in the Ca^2+^‐permeable AMPAR population due to AMPAR subunit dysregulation. Here, we provide the first comparative quantitative assessment of the expression profile of AMPAR subunit transcripts, using BaseScope, in post‐mortem lower motor neurons (spinal cord, anterior horn), upper motor neurons (motor cortex) and neurons of the pre‐frontal cortex in sALS and fALS due to mutations in *SOD1* and *C9orf72*. Our data indicated that AMPAR dysregulation is prominent in lower motor neurons in all ALS cases. However, sALS and mutant *C9orf72* cases exhibited *GluA1* upregulation whereas mutant SOD1 cases displayed *GluA2* down regulation. We also showed that sALS cases exhibited widespread AMPAR dysregulation in the motor and pre‐frontal cortex, though the exact identity of the AMPAR subunit being dysregulated was dependent on brain region. In contrast, AMPAR dysregulation in mutant *SOD1* and *C9orf72* cases was restricted to lower motor neurons only. Our data highlight the complex dysregulation of AMPAR subunit expression that reflects both converging and diverging mechanisms at play between different brain regions and between ALS cohorts. © 2019 Authors. *Journal of Pathology* published by John Wiley & Sons Ltd on behalf of Pathological Society of Great Britain and Ireland.

## Introduction

Amyotrophic lateral sclerosis (ALS) is a rapidly progressive and invariably fatal neurodegenerative disease characterised by degeneration of motor neurons of the brain and spinal cord. The last 10 years has seen considerable progress in the genetic understanding of ALS with over 40 associated genes known to harbour genetic mutations associated with the disease. However, only a small proportion of total ALS cases (5–10%) are linked to hereditary mutations and the proportion of apparently sporadic ALS (sALS) cases having a genetic basis remains ∼10% 1, 2. In this regard, modelling sALS has been challenging and the majority of ALS research has been conducted on models based on genetic mutations including the *C9orf72* repeat expansion (*C9orf72* ^*RE*^) mutation and mutations to *SOD1*, which represent the two most frequent known familial and sporadic ALS mutations 1, 2. Whilst many mutations share general pathological features with sALS, including TDP‐43 pathology 3, there is an appreciation that the diversity of mutated genes are also likely to reflect diverse pathways and mechanisms that underlie the degeneration of motor neurons in ALS 4, 5. The extrapolation and relevance of how these pathways converge and diverge between the multiple known familial causes of ALS and sporadic forms of ALS is largely unknown.

Glutamate‐mediated excitotoxicity has been a major hypothesis in ALS motor neuron degeneration for ∼25 years, with α‐amino‐3‐hydroxyl‐5‐methyl‐4‐isoxazole‐propionate receptors (AMPARs) emerging as a likely candidate for glutamate receptor‐mediated excitotoxicity 6. Crucially, glutamate‐gated AMPAR ion channels are composed of four potential subunits, GluA1–4, each of which genetically encode Ca^2+^‐permeable subunits 7. The GluA2 subunit is however almost uniformly subject to post‐transcriptional RNA editing such that insertion of edited GluA2 subunits into the AMPAR complex confers Ca^2+^‐impermeability to the ion channel. Dysregulation of AMPAR subunits to generate increased Ca^2+^‐permeable AMPAR populations is thought to contribute to excitotoxicity in ALS motor neurons 8, 9, 10, 11, 12, 13, 14, 15. Indeed, recently we showed that *C9orf72* ^*RE*^ patient motor neurons displayed a vulnerability to AMPAR‐mediated excitotoxicity due to a *C9orf72* ^*RE*^‐dependent increase in Ca^2+^‐permeable AMPAR expression through an abnormal increase in GluA1 subunit expression 15. However, altered AMPAR properties were not observed in human cortical neurons suggesting that the *C9orf72* ^*RE*^ mutation imparts a selective AMPAR‐associated mechanism of excitotoxicity onto motor neurons 15. The degree to which mechanisms are conserved across specific brain areas and furthermore other ALS patients with different familial and sporadic aetiologies remains to be clarified.

Noting sporadic and *C9orf72* ^*RE*^ ALS patients, but not *SOD1* mutation patients, typically exhibit shared TDP‐43 pathology 16 we have therefore, for the first time, compared the regional expression of AMPARs in sALS patients together with patients with *SOD1* (I114T) and *C9orf72* ^*RE*^ mutations. To accomplish this we used a high‐resolution *in situ* hybridisation technique, BaseScope, to systematically characterise the expression of AMPAR subunit transcripts at the single‐cell level in post‐mortem spinal cord (anterior horn), prefrontal cortex and motor cortex from the different ALS cohorts with respect to age‐ and sex‐matched controls with no clinical or pathological evidence of neurological disease. Furthermore, we have used human pluripotent stem cell technology to examine the degree of *GluA2* RNA editing within sALS patient‐derived neurons. Our data implicate notable regional AMPAR subunit dysregulation across all brain regions examined in sALS patients and a restriction of AMPAR subunit dysregulation to the spinal cord in *SOD1* (I114T) and *C9orf72* ^*RE*^ patients.

## Materials and methods

### Case identification and ethics

ALS post‐mortem samples were obtained from the Medical Research Council (MRC) Edinburgh Brain Bank and had separately undergone whole genome sequencing for genetic identification 2. Our study used three separate cases for each of *C9orf72* repeat expansion, sporadic and *SOD1* ALS cases. Age and sex‐matched control cases with respect to the ALS cases, exhibited no evidence of neurodegenerative disease pathology and were obtained from the Edinburgh Sudden Death Brain Bank. All clinical data were collected as part of the Scottish Motor Neurone Disease Register and Care Audit Research and Evaluation for Motor Neurone Disease platform (Ethics approval from Scotland A Research Ethics Committee 10/MRE00/78 and 15/SS/0216) and all patients consented to the use of their data during life. All post‐mortem tissue was collected via the Edinburgh Brain Bank (Ethics approval from East of Scotland Research Ethics Service, 16/ES/0084) in line with the Human Tissue (Scotland) Act (2006). Use of human tissue for post‐mortem studies was reviewed and approved by the Edinburgh Brain Bank ethics committee and the Academic and Clinical Central Office for Research and Development medical research Ethics Committee.

### Histology and neuropathological assessment

Brain tissue was taken post‐mortem from standardised Brodmann areas (BA), BA4 and BA9 and spinal cord and fixed in 10% formalin for a minimum of 72 h. Tissue was dehydrated in an ascending alcohol series (70–100%) followed by three successive 4 h washes in xylene. Three successive 5 h paraffin wax embedding stages were performed followed by cooling and sectioning of the FFPE (formalin‐fixed paraffin embedded) tissue on a Leica microtome into 4 μm thick sections that were collected on Superfrost microscope slides. Sections were dried overnight at 40 °C and immunostaining was performed, following epitope retrieval in citric acid buffer (pH 6) in a pressure cooker for 30 min, using the Novolink Polymer detection system with the Proteintech (Manchester, UK) anti‐phospho(409–410)‐TDP‐43 antibody at a 1 in 1000 dilution and Abcam (Cambridge, UK) anti‐glutamate receptor 1 antibody (ab32436) at a 1 in 50 dilution (both incubated for 30 min at room temperature). Counterstaining was performed using DAB chromogen counterstained with haematoxylin, according to standard operating procedures. TDP‐43 pathology was graded semi‐quantitatively by two independent pathologists, using the following descriptive scoring system: (1) no TDP‐43 pathology; (2) mild TDP‐43 pathology (up to 5 affected cells in at least one ×40 high power field (HPF) out of three HPFs examined); (3) moderate TDP‐43 pathology (5–15 affected cells in at least one ×40 HPF out of three HPFs examined); severe TDP‐43 pathology (>15 cells affected in at least one ×40 HPF out of three HPFs examined). Assessors were blinded to all demographic and clinical information. Motor neurons were identified based on anatomical location within the spinal cord and according to established neuropathological criteria including size and morphology.

### BaseScope analysis

FFPE tissue was sectioned at 4 μm thickness on to Superfrost slides. BaseScope reagents (Advanced Cell Diagnostics Inc., Newark, CA, USA) were used following the manufacturer's guidelines according to the original protocol. In brief, following deparaffinisation, tissue sections were incubated with hydrogen peroxide for 10 min at room temperature and target antigen retrieval was performed by submerging slides in BaseScope ×1 target retrieval reagent at 99 °C in a Braun Multiquick FS 20 steamer for 15 min. The tissue was then permeabilised using BaseScope protease III at 40 °C for 30 min. Probe hybridisation was then performed by incubating the slides with 4 drops of custom designed BaseScope probe, negative control probe (dihydrodipicolinate reductase, *DapB*) or positive control (peptidyl‐prolyl cis‐trans isomerase B, *PPIB*) probe for 2 h at 40 °C. Following successive probe amplification steps, transcripts were detected using the BaseScope RED detection kit and slides were counterstained using haematoxylin and lithium carbonate. The slides were then cleared in xylene and mounted with a 24 × 50 mm coverslip using two drops of VectaMount mounting medium. Sections were then imaged at ×20 objective magnification on a NanoZoomer slide scanner. An unsharp mask in Photoshop was equally applied to all BaseScope images to improve transcript signal visibility in panels, all quantification was performed using direct visualisation under the microscope to facilitate accurate transcript resolution. Motor neurons were identified according to established neuropathological criteria including size and morphology. Number of spots/per cell was counted for experimental GluA1–4 and PPIB probes individually. Due to the sensitivity and specificity of BaseScope, one red dot corresponds to one transcript, therefore transcript copy number values were taken to be absolute. Counts were plotted as individual transcripts per cell. GluA^TOTAL^ for each patient was calculated by summing the subunits GluA1–4. The amount of Ca^2+^‐permeable AMPAR was estimated by taking a ratio of (GluA1 + GluA3 + GluA4)/GluA^TOTAL^. Positive (PPIB) and negative (DapB; a bacterial gene that is not expressed in human tissue) control probes were also quantified (number of spots/neuron) in the same way as each of our experimental probes (GluA1–4). We did not observe differences in *PPIB* expression between controls and cases across all sampled regions (Figures 2A, 4A and 6A) and there was no expression of DapB in any of the material examined (data not shown).

### Induced pluripotent stem cell derived motor neurons and *GluA2* editing

Dermal fibroblasts from sALS patient and control individuals were obtained under full Ethical/Institutional Review Board approval at the University of Edinburgh. Fibroblasts were reprogrammed to iPSCs using episomal vectors expressing OCT4, SOX2, C‐MYC and KLF4. iPSCs were maintained in Matrigel (BD Biosciences, San Jose, CA, USA)‐coated plastic dishes in E8 medium (Life Technologies, Renfrew, UK) at 37 °C and 5% CO_2_. Motor neurons were differentiated according to a protocol detailed previously 15. RNA editing of the *GluA2* subunit was performed exactly as detailed previously 15.

### Statistical analysis

The relative abundance of transcripts was quantified by assessing the mean (±SD) number of red dots per cell across 10 randomly identified cells in three randomly generated fields of view (1 mm^2^) for each case. Cells within each field of view were randomly identified and to prevent bias the assessor was blinded to all clinical and identifiable data and to the GluA1–4 subunit being assessed. Cases were compared using one‐way ANOVA with Bonferroni's *post hoc* test to account for multiple comparisons. Significance values; *p < 0.05; **p < 0.01; ***p < 0.001. Fold‐change compared to *GluA2* expression was performed by correcting each case to its age and sex‐matched control.

## Results

This study has employed a highly sensitive *in situ* hybridisation method (BaseScope) to examine the expression of AMPAR subunit mRNA transcripts (*GluA1–4*) at a single‐cell level in post‐mortem spinal cord (anterior horn cells), motor cortex (BA4) and prefrontal cortex (BA9) tissue from sporadic, *SOD1* I114T and *C9orf72* ^*RE*^ ALS patients (three patients for each ALS cohort). AMPAR expression in ALS post‐mortem tissue was compared to post‐mortem tissue from three age‐ and sex‐matched controls with no clinical history or pathological detection of neurodegenerative disease (neuropathological data summarised in Table 1 and cohort demographics in Table 2).

**Table 1:** Summary of neuropathological assessment of cases

	TDP‐43 immunostaining			p62 immunostaining
Case	Spinal cord	Prefrontal cortex	Motor cortex	
1 – sALS	Anterior horn cells lost. Neuronal inclusions highlighted by TDP‐43, p62 and Ubiquitin were seen. GFAP highlighted reactive gliosis	Mild* abundance of neuronal and glial TDP‐43 inclusions	Mild* abundance of neuronal and glial TDP‐43 inclusions	There was no evidence of non‐TDP‐43 related p62 staining
2 – sALS	Anterior horn cells lost. The few remaining anterior horn cells contained inclusions which immunoreacted for p62, TDP‐43 and Ubiquitin. In addition, there was extensive glial pathology highlighted by TDP‐43 and p62	Mild* abundance of neuronal and glial TDP‐43 inclusions	Mild* abundance of neuronal and moderate* abundance of glial TDP‐43 inclusions	There was no evidence of non‐TDP‐43 related p62 staining
3 – sALS	Anterior horn cells lost. Cytoplasmic inclusions were immunoreactive for TDP‐43 and were seen in residual anterior horn cells throughout the spinal cord	Mild* abundance of neuronal and glial TDP‐43 inclusions	Mild* abundance of neuronal and glial TDP‐43 inclusions	There was no evidence of non‐TDP‐43 related p62 staining
1 – SOD1	Anterior horn cells lost. No TDP‐43 inclusions were seen within the neocortical ribbon, brain stem nuclei or within residual anterior horn cells	No evidence of neuronal or glial TDP‐43 inclusions	No evidence of neuronal or glial TDP‐43 inclusions	There was no evidence of non‐TDP‐43 related p62 staining
2 – SOD1	Anterior horn cells lost. No TDP‐43 inclusions were seen within the neocortical ribbon, brain stem nuclei or within residual anterior horn cells	No evidence of neuronal or glial TDP‐43 inclusions	No evidence of neuronal or glial TDP‐43 inclusions	There was no evidence of non‐TDP‐43 related p62 staining
3 – SOD1	Anterior horn cells lost. No TDP‐43 inclusions were seen within the neocortical ribbon, brain stem nuclei or within residual anterior horn cells	No evidence of neuronal or glial TDP‐43 inclusions	No evidence of neuronal or glial TDP‐43 inclusions	There was no evidence of non‐TDP‐43 related p62 staining
1 – Control	No pathology	No pathology	No pathology	No pathology
2 – Control	No pathology	No pathology	No pathology	No pathology
3 – Control	No pathology	No pathology	No pathology	No pathology
1 – *C9orf72*	Anterior horn cells lost. TDP‐43 inclusions were noted within residual anterior horn cells	Mild* abundance of neuronal and glial TDP‐43 inclusions	Mild* abundance of neuronal and glial TDP‐43 inclusions	There was abundant p62 pathology within the amygdala, hippocampus and cerebellum
2 – *C9orf72*	Anterior horn cells lost. TDP‐43 inclusions were noted within residual anterior horn cells	Mild* abundance of neuronal and moderate* abundance of glial TDP‐43 inclusions	Mild* abundance of neuronal and moderate* abundance of glial TDP‐43 inclusions	There was striking p62 immunoreactivity throughout the neocortex and within the hippocampus, particularly the dentate gyrus. In addition, p62 expression was noted within the cerebellar cortex particularly within the granule cell layer
3 – *C9orf72*	Anterior horn cells lost. TDP‐43 inclusions were noted within residual anterior horn cells	Mild* abundance of neuronal and no evidence of glial TDP‐43 inclusions	Moderate* abundance of neuronal and glial TDP‐43 inclusions	There was abundant p62 pathology within the amygdala, hippocampus and cerebellum

Abundance of pathological cortical TDP‐43 inclusions (determined using an antibody against the pathologically phosphorylated form of TDP‐43) are scored as follows: *Mild (<5 affected cells in at least one ×20 field of view per section); *moderate (5–15 affected cells in at least one ×20 field of view per section); *severe (>15 cells affected in at least one ×20 field of view per section). Spinal cord TDP‐43 inclusion assessment is descriptive due to differences in abundance of anterior horn cells (markedly reduced in number in ALS spinal cord).

**Table 2:** Patient and control cohort characteristics

Patient	Sex	Age	Disease duration
1 – sALS	M	54	66
2 – sALS	M	70	16
3 – sALS	F	68	24
1 – *C9orf72*	F	62	97
2 – *C9orf72*	F	63	109
3 – *C9orf72*	F	63	33
1 – *SOD1*	M	64	67
2 – *SOD1*	F	68	127
3 – *SOD1*	F	56	98
1 – Control	M	58	N/A
2 – Control	M	65	N/A
3 – Control	F	68	N/A

Age indicates age at death; disease duration indicates months from symptom‐onset to death.

### Control data

Our positive control slides localising *PPIB* transcripts showed no observable difference in *PPIB* expression between controls and cases across all sampled regions. No staining was seen in our negative control slides. By using both positive and negative controls, we have confidence that our cases are directly comparable in a robust and reproducible manner. Our PPIB data is described and presented for each of the brain regions in which AMPARs subunits were investigated.

### AMPAR subunit expression is altered in sporadic, *C9orf72* repeat expansion and mutant *SOD1* ALS motor neurons

**Figure 1:**
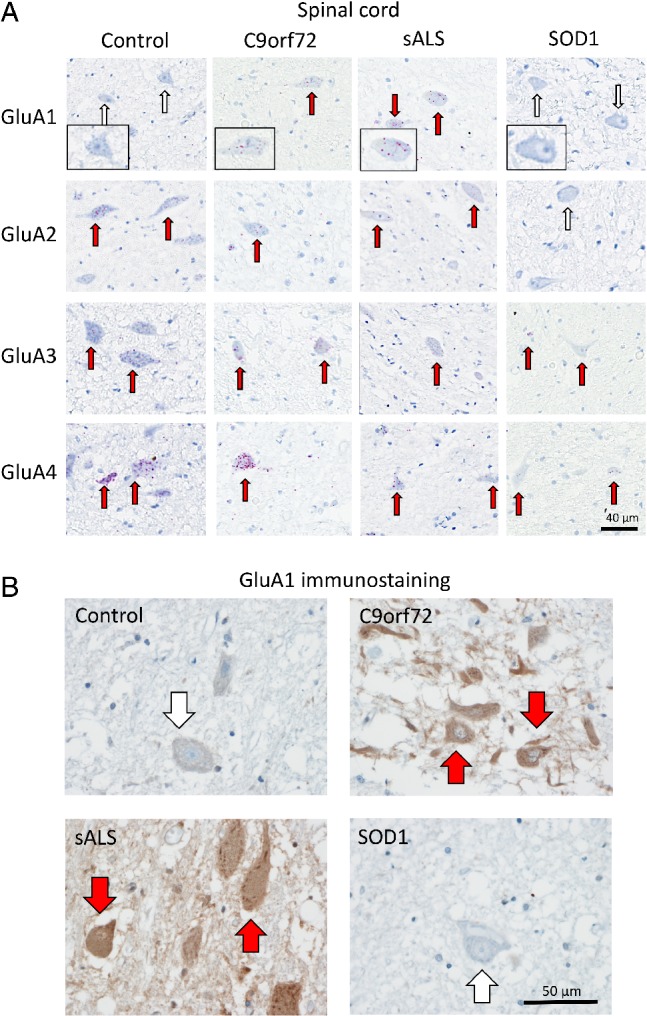
Characterisation of AMPAR subunit mRNA expression in human post‐mortem anterior horn cells. (A) Representative images of BaseScope *in situ* hybridisation demonstrating quantifiable expression of *GluA1*, *GluA2*, *GluA3* and *GluA4* AMPAR subunit mRNA in anterior horn cells of control, *C9orf72* ^*RE*^, sALS and *SOD1* I114T ALS cases. The red dots (*arrows*) represent individual detected mRNA molecules. Red arrows highlight cells with mRNA expression and white arrows indicate low/no expression. Note the lack of *GluA1* transcripts within control patient spinal cord cells and the presence of *GluA1* within sALS and *C9orf72* ^*RE*^ patient cells. Note the lack of *GluA2* (and *GluA1*) in *SOD1* I114T patient cells. Scale bar, 20 μm. (B) Representative images of IHC staining for the GluA1 subunit within the anterior horn of the spinal cord of control individuals as well as sALS, *C9orf72* and *SOD1* cases. White arrows indicate anterior horn cells (spinal motor neurons) with no staining and red arrows indicate anterior horn cells (spinal motor neurons) with IHC staining for GluA1. Indicating increased levels of GluA1 subunit (protein) detected by IHC.

**Figure 2:**
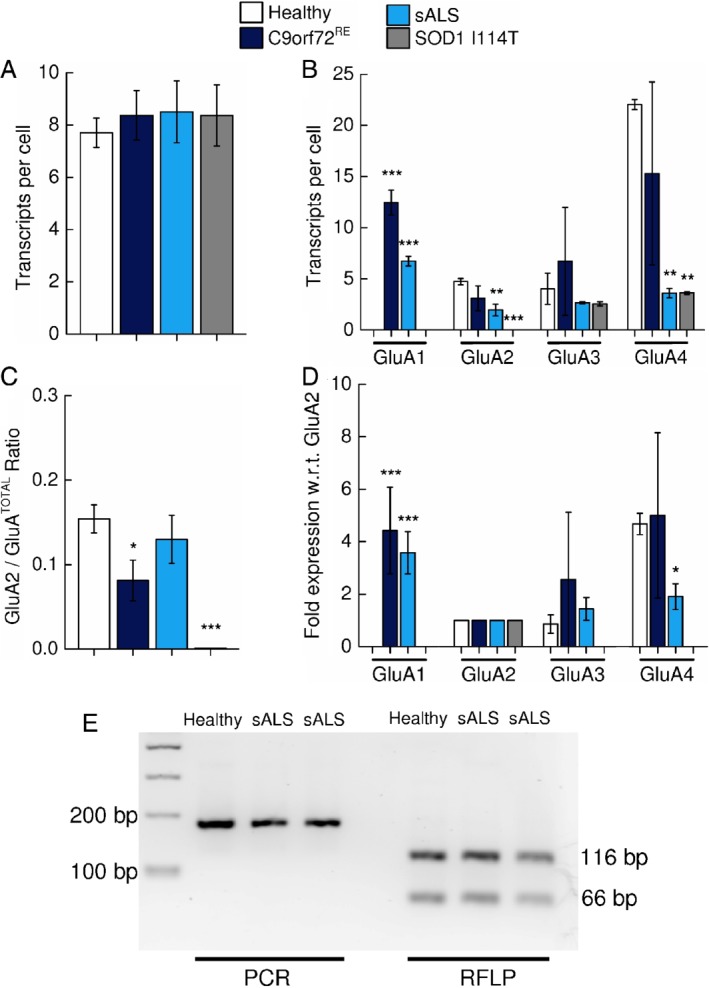
Quantification of cellular AMPAR subunit mRNA transcript expression within anterior horn cells. (A) Mean (±SD) number of positive‐control *PPIB* mRNA transcripts per cell (dots per cell = number of mRNA transcripts per cell) within the anterior horn cells of sALS, *C9orf72* ^*RE*^, *SOD1* I114T and control individuals. (B) Mean (±SD) number of *GluA1*, *GluA2*, *GluA3* and *GluA4* AMPAR subunit mRNA transcripts per cell within anterior horn cells of three control, *C9orf72* ^*RE*^, sALS and *SOD1* I114T ALS cases. Note the detection of *GluA1* transcripts in sALS and *C9orf72* ^*RE*^ patients contrasting against the absence of *GluA1* transcripts in control patients. Note the absence of *GluA2* (and *GluA1*) transcripts in *SOD1* I114T patients. *C9orf72* ^*RE*^ repeat expansion patient data has been previously presented in 14. *PPIB* and AMPAR data from each patient was a mean determined from a total of 30 cells from three randomly generated fields of view (10 cells/field of view). *C9orf72* ^*RE*^ patient data has been previously presented in 15. (C) Mean (±SD) relative expression of *GluA2* subunit mRNA expressed as a percentage with respect to total AMPAR subunit transcripts (*GluA2*/*GluA* ^TOTAL^). (D) Mean (±SD) normalised fold‐expression of Ca^2+^‐permeable *GluA1*, *GluA3* and *GluA4* AMPAR subunit mRNA with respect to (w.r.t.) *GluA2* transcripts. (E) Representative gel picture showing efficient GluA2 Q→R RNA editing in week 3 cultures in sALS and control individual lines. RFLP analysis is on the right, where efficient *GluA2* RNA editing results in RFLP amplicons of 116 and 66 bp. A band at 81 bp would be observed with inefficient GluA2 subunit editing. The PCR product for GluA2 is shown on the left indicating GluA2 expression. Statistical analysis performed with one‐way ANOVA with Bonferroni's *post hoc* correction. Statistical significance highlighted is with respect to control individuals. We calculated GluA^TOTAL^ for each patient by summing the subunits *GluA1–4*.

Previously we have shown transcriptional upregulation of the Ca^2+^‐permeable GluA1 AMPAR subunit in *C9orf72* ^*RE*^ patient post‐mortem anterior horn cells (spinal motor neurons) compared to controls 15. We and others have shown that *GluA1* mRNA expression in adult spinal cord motor neurons is absent or negligible 8, 9, 15, 17, 18. To determine if this transcriptional dysregulation of AMPAR subunit is *C9orf72* ^*RE*^ mutation specific, we extended our analysis to sALS and *SOD1* I114T spinal motor neurons. Equivalent to *C9orf72* ^*RE*^ patients, our data from sALS patients demonstrate an increased *GluA1* mRNA abundance in the anterior horn cells of the spinal cord with respect to controls which displayed no measurable level of *GluA1* transcripts, whilst displaying equivalent expression of the positive control transcript, *PPIB* (Figures 1A and 2A,B). For ease of comparison, we have presented the raw quantified AMPAR subunit transcript data previously published for *C9orf72* ^*RE*^ patient post‐mortem anterior horn cells (and respective controls; 15, Figure 2B). However, SOD1 I114T patient samples did not express *GluA1* transcripts in anterior horn motor neurons (Figures 1 and 2B. These data were further confirmed by immunohistochemical (IHC) staining for the GluA1 subunit (protein) in post‐mortem spinal cord tissue and finding increased staining for GluA1 in the anterior horn cells (spinal motor neurons) of *C9orf72* ^*RE*^ and sALS cases, with no staining for GluA1 in control individuals and *SOD1* cases (Figure 1B). Given that TDP‐43 pathology is a common pathological signature observed in *C9orf72* ^*RE*^ and sALS and absent in mutant SOD1 patient post‐mortem brain tissues 16, 19, these data support an association between TDP‐43 pathology and the transcriptional dysregulation of *GluA1* in spinal cord motor neurons. Indeed, prominent pathological TDP‐43 aggregation was observed in the anterior horn cells of sALS and *C9orf72* ^*RE*^ patients but was not present in *SOD1* I114T patient samples (Table 1).

The GluA2 subunit typically controls and confers Ca^2+^‐impermeability onto the AMPAR ion channel 7 and its reduced expression (which would result in calcium permeability) has been previously highlighted as a factor that may underpin increased vulnerability of ALS motor neurons to glutamate excitotoxicity 9, 13, 20. However, the data in our study demonstrate that, in direct contrast to *C9orf72* ^*RE*^, sALS patients and controls, *GluA2* mRNA expression was not detectable in *SOD1* I114T anterior horn cells indicating exclusive expression of Ca^2+^‐permeable AMPARs. We did not examine protein expression of the GluA2 (and GluA3 and GluA4) subunits given the inability of antibodies to discriminate between these AMPAR subunits. To assess the impact of alterations in AMPAR subunit expression upon the potential functional Ca^2+^‐permeable versus Ca^2+^‐impermeable AMPAR identity in each of the ALS patients and control individuals, we initially calculated the ratio of *GluA2* subunit mRNA expression with respect to total AMPAR subunit expression (*GluA2/GluA* ^*TOTAL*^) for each ALS group, where 0 and 1 reflect purely Ca^2+^‐permeable and Ca^2+^‐impermeable AMPARs populations respectively (Figure 2C). Surprisingly, despite an abnormal increase in *GluA1* transcript level, we found that sALS motor neurons exhibited a *GluA2/GluA* ^*TOTAL*^ ratio that was comparable to that of control individuals. However, *C9orf72* ^*RE*^ patients had significantly lower *GluA2/GluA* ^*TOTAL*^, which was therefore consistent with an increased Ca^2+^‐permeable AMPAR population.

To understand how expression of *GluA1*, *GluA3* and *GluA4* subunits individually contribute to the predicted total Ca^2+^‐permeable versus Ca^2+^‐impermeable AMPAR identity we normalised each subunit data relative to *GluA2* subunits (Figure 2D). This analysis confirmed that *GluA1* expression was substantially elevated in anterior horn cells of both sALS and *C9orf72* ^*RE*^ with respect to control individuals. However, considered together with the *GluA2/GluA* ^*TOTAL*^ data displayed in Figure 2C, the impact of increased *GluA1* expression in sALS patients appears to be compensated by a decrease in the relative expression of *GluA4* transcripts, which was significantly reduced with respect to control cases. For *C9orf72* ^*RE*^ patients, the relative expression data for *GluA3* and *GluA4* subunits were not significantly changed with respect to control patient data. The large standard deviation for the relative expression data of *GluA3* and *GluA4* subunits is due to a high degree of reciprocal variability between patients (Table 3). The abnormal increase in *GluA1* in *C9orf72* ^*RE*^ patient motor neurons therefore appears to be uncompensated leading to an increase in Ca^2+^‐permeable AMPARs.

**Table 3:** Reciprocal expression of *GluA3* and *GluA4* in *C9orf72* ^*RE*^ spinal cord motor neurons

	GluA3		GluA4	
Case	Mean	SD	Mean	SD
1 – C9orf72	4.6	0.84	19.9	5.09
2 – C9orf72	2.8	0.42	21	4.24
3 – C9orf72	12.7	2.21	5	0.82
1 – Control	2.8	1.32	3.7	2.00
2 – Control	2.6	1.78	3.1	1.20
3 – Control	2.6	1.51	4	1.83
1 – SOD1	2.5	1.58	3.5	1.08
2 – SOD1	2.4	1.43	3.6	1.35
3 – SOD1	2.8	2.25	3.8	1.03
1 – sALS	4.7	0.48	22.6	4.20
2 – sALS	5.1	0.88	21.7	4.16
3 – sALS	2.3	0.67	21.8	6.65

Mean and SD of the number of *GluA3* and *GluA4* mRNA transcripts in each case, demonstrating reciprocal expression profiles. Light shading – higher expression; Dark shading – lower expression; No shading – no difference in expression.

Sporadic ALS post‐mortem spinal cord motor neurons also exhibit inefficient RNA editing of the *GluA2* subunit 10. This appears to be an sALS‐specific feature since mutant *SOD1* post‐mortem motor neurons 20 and *C9orf72* ^*RE*^ patient‐derived motor neurons 15 do not exhibit inefficient *GluA2* editing. Because BaseScope probe design is not yet conducive for comparison of unedited versus edited *GluA2* levels (essentially a single base pair substitution) we examined the efficiency of *GluA2* editing in enriched cultures of motor neurons differentiated *in vitro* from induced pluripotent stem cells obtained from a sALS patient, as described previously 15. We employed restriction fragment length polymorphism (RFLP) analysis using the restriction enzyme Bbv1 and this indicated that *GluA2* editing is highly efficient in sALS patient‐derived motor neurons (Figure 2E).

### Ca^2+^‐permeable AMPAR subunit expression is elevated in the motor cortex of sporadic ALS patients

**Figure 3:**
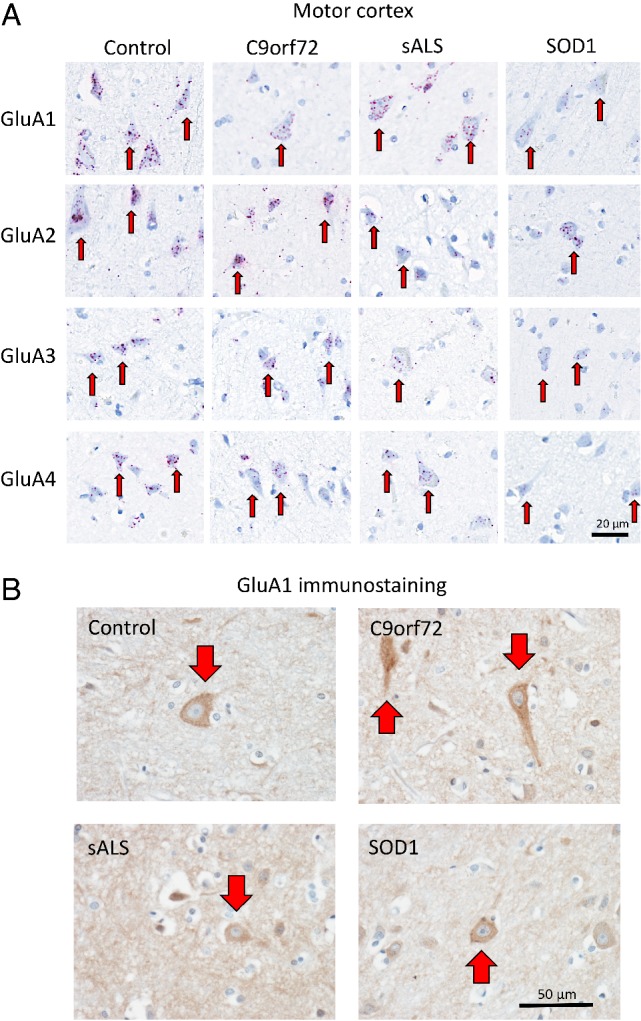
Characterisation of AMPAR subunit mRNA expression in human post‐mortem motor cortex tissue. Data as described in Figure 1, but for the motor cortex (BA4). Note the increased detection of Ca^2+^‐permeable *GluA1*, *GluA3* and *GluA4* AMPAR subunit mRNA transcripts relative to *GluA2* transcripts in sALS patients. (B). Representative images of IHC staining for GluA1 subunit within the motor cortex of control individuals as well as sALS, *C9orf72* and *SOD1* cases. Red arrows indicate cortical motor neurons with positive IHC staining for GluA1. Indicating no discernible difference in levels of GluA1 subunit (protein) detected by IHC.

**Figure 4:**
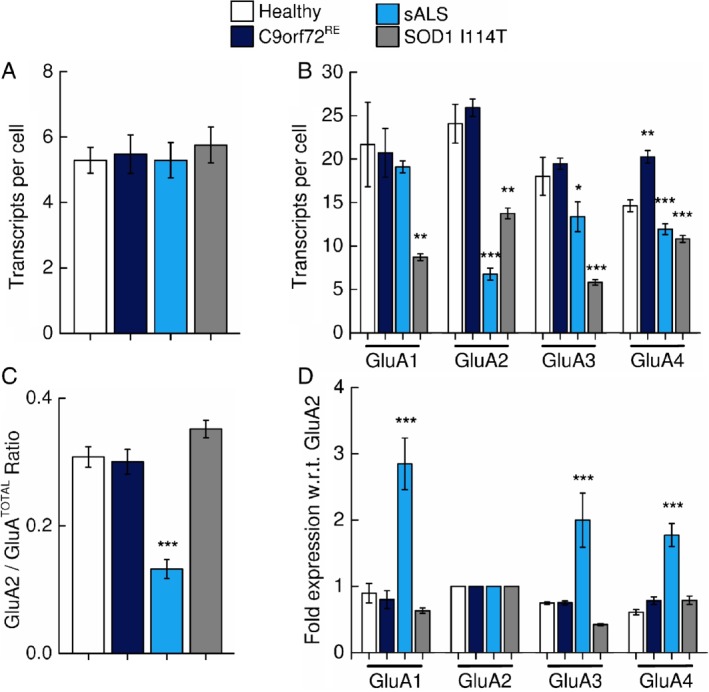
Quantification of cellular AMPAR subunit mRNA transcript expression within motor cortex tissue. (A) Mean (±SD) number of positive‐control *PPIB* and (B), mean (±SD) AMPAR subunit mRNA transcripts per cell as described in Figure 1, but for the motor cortex. (C) Mean (±SD) relative expression of *GluA2* subunit mRNA expressed as a percentage with respect to total AMPAR subunit transcripts (*GluA2*/*GluA* ^TOTAL^). (D) The mean (±SD) normalised fold‐expression of Ca^2+^‐permeable *GluA1*, *GluA3* and *GluA4* AMPAR subunit mRNA with respect to (w.r.t.) *GluA2* transcripts. Note the increased detection of Ca^2+^‐permeable *GluA1*, *GluA3* and *GluA4* AMPAR subunit mRNA transcripts relative to *GluA2* transcripts in sALS patients. Statistical analysis performed with one‐way ANOVA with Bonferroni's *post hoc* correction. Statistical significance highlighted is with respect to control individuals.

The principle feature of ALS is degeneration of both lower and upper motor neurons. We therefore examined whether expression of AMPAR subunit transcripts in cortical motor neurons (upper motor neurons, BA4) are altered in sALS, *C9orf72* ^*RE*^ and *SOD1* I14T patients (Figure 3A). Motor cortex samples displayed equivalent staining for PPIB (Figure 4A) and subsequent quantification of AMPAR subunit mRNA transcripts revealed the variable expression of all transcripts in all ALS cases examined (Figure 4B). For the first time, we showed that *C9orf72* ^*RE*^ patients expressed comparable levels of AMPAR subunit expression with respect to control individuals in cortical motor neurons with the exception of a modest increase in levels of the *GluA4* subunit transcript. Interestingly, we observed a profound reduction of *GluA2* subunit expression in sALS cases and modest reductions in *GluA3* and *GluA4* subunit expression. In contrast, *SOD1* I114T patients displayed reduced expression of each of the AMPAR subunits when compared to control individuals (Figure 4B).

To estimate the levels of Ca^2+^‐permeable AMPARs in cortical motor neurons, we assessed *GluA2/GluA* ^*TOTAL*^, as discussed for anterior horn cells. Importantly, *GluA2/GluA* ^*TOTAL*^ was found to be substantially reduced in sALS, but not *C9orf72* ^*RE*^ or *SOD1* I114T, patients compared to control motor cortex (Figure 4C). Therefore, despite sALS and *C9orf72* ^*RE*^ patients both exhibiting TDP‐43 pathology (Table 1), sALS neurons of the motor cortex only exhibit a transcript profile consistent with an increased Ca^2+^‐permeable AMPAR population.

To understand this change in AMPAR dysregulation in sALS patients with respect to control individuals, we then normalised each AMPAR subunit data relative to *GluA2* (Figure 4D). Notably, we observed a 2‐ to 3‐fold significant increase in the levels for all Ca^2+^‐permeable subunits (*GluA1*, *GluA3* and *GluA4*) with respect to controls. This is a reflection of normalisation to *GluA2*, which is substantially reduced with respect to control individuals. Inspection of the raw transcript data in Figure 4B indicates a substantial reduction of *GluA2*, but not *GluA1*, *GluA3* or *GluA4* transcripts with respect to control data. These data indicate that the motor cortex of sALS patients exhibit a transcriptional downregulation of the *GluA2* subunit, which is consistent with a Ca^2+^‐permeable AMPAR phenotype.

### Relative *GluA1* AMPAR subunit expression is elevated in the prefrontal cortex of sporadic ALS patients

**Figure 5:**
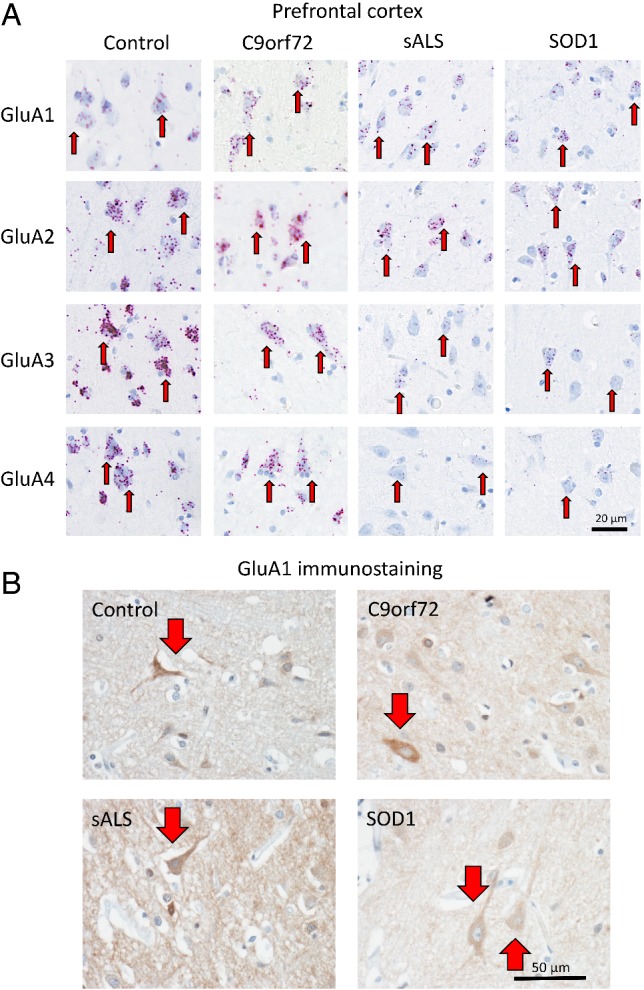
Characterisation of AMPAR subunit mRNA expression in human post‐mortem prefrontal cortex tissue. (A) Data as described in Figure 1, but for the prefrontal cortex. Note the increased detection of Ca^2+^‐permeable *GluA1* AMPAR subunit mRNA transcripts relative to *GluA2* transcripts in sALS patients. (B) Representative images of IHC staining for GluA1 subunit within the prefrontal cortex of control individuals as well as sALS, *C9orf72* and *SOD1* cases. Red arrows indicate cortical neurons with positive IHC staining for GluA1. Images indicate a possible mild increase in GluA1 staining in the sALS and decrease in SOD1 cases consistent with the mRNA transcript abundance in (A), however, there was no definite discernible difference in levels of GluA1 subunit detected.

**Figure 6:**
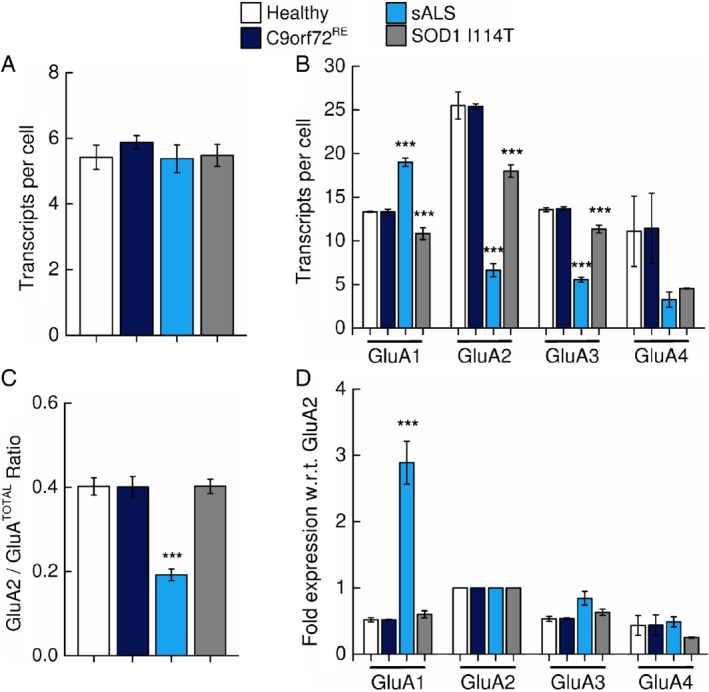
Quantification of cellular AMPAR subunit mRNA transcript expression within prefrontal cortex tissue. (A) Mean (±SD) number of positive‐control *PPIB* and (B), mean (±SD) AMPAR subunit mRNA transcripts per cell as described in Figure 1, but for the prefrontal cortex. *C9orf72* ^*RE*^ repeat expansion patient data has been presented previously in 15. (C) Mean (±SD) relative expression of *GluA2* subunit mRNA expressed as a percentage with respect to total AMPAR subunit transcripts (*GluA2*/*GluA* ^TOTAL^). (D) Graph showing the mean (±SD) normalised fold‐expression of Ca^2+^‐permeable *GluA1*, *GluA3* and *GluA4* AMPAR subunit mRNA with respect to (w.r.t.) *GluA2* transcripts. Note the increased detection of Ca^2+^‐permeable *GluA1* AMPAR subunit mRNA transcripts relative to *GluA2* transcripts in sALS patients. Statistical analysis performed with one‐way ANOVA with Bonferroni's *post hoc* correction. Statistical significance highlighted is with respect to control individuals.

To determine whether AMPAR subunit dysregulation is associated with upper and lower motor neurons or, non‐motor neurons in other brain regions also affected by TDP‐43 pathology in ALS patients we examined AMPAR subunit expression in the post‐mortem prefrontal cortex (BA9) of sALS, *C9orf72* ^*RE*^ and *SOD1* I114T patients (Figure 5A). Cellular quantification of positive control *PPIB* (Figure 6A) and AMPAR subunit transcripts was performed as for sALS and *SOD1* I114T patients (Figure 6B). Previously published quantified *C9orf72* ^*RE*^ AMPAR subunit data for the prefrontal cortex 15 is presented for comparison (Figure 6B). All AMPAR subunit transcripts were detected in each of the cases and control individuals. AMPAR subunits were not extensively altered in prefrontal cortex neurons in *C9orf72* ^*RE*^ and SOD1 I114T cases. However, in sALS cases, *GluA2* and *GluA3* transcripts were reduced substantially and there was a significant upregulation of *GluA1* mRNA (Figure 6B). These data were further confirmed by IHC staining for the GluA1 subunit (protein) in post‐mortem prefrontal cortex, demonstrating a possible increase in staining for GluA1 in the neurons of sALS cases compared to controls (Figure 5B).

Strikingly, the *GluA2/GluA* ^*TOTAL*^ ratio in the prefrontal cortex of sALS patients was lower than in controls, therefore suggesting a substantially larger Ca^2+^‐permeable AMPAR population (Figure 6C). *GluA2/GluA* ^*TOTAL*^ prefrontal cortex data generated for *C9orf72* ^*RE*^ and *SOD1* I114T patients revealed no significant difference in the *GluA2/GluA* ^*TOTAL*^ ratio (Figure 6C). Of interest, we additionally note that for the prefrontal cortex of control individuals that *GluA2/GluA* ^*TOTAL*^ ratios are significantly higher than that of the motor cortex (*p* < 0.01, unpaired *t*‐test) and anterior horn cells (*p* < 0.001, unpaired *t*‐test), indicating the different transcriptional regulation of AMPAR subunits between different brain regions.

We then examined how the shift in *GluA2/GluA* ^*TOTAL*^ ratio in sALS prefrontal cortex with respect to control was underpinned by regulation of individual AMPAR subunits by normalisation of each subunit data to *GluA2* for each patient (Figure 6D). *GluA1* transcript expression was found robustly enhanced in the post‐mortem motor cortex of sALS patient versus control cases, whilst the relative expression of *GluA3* and *GluA4* was not significantly different from that in control individuals. The dysregulation of *GluA1* subunit expression in the prefrontal cortex of sALS patients is therefore likely to underpin the observed reduction in *GluA2/GluA* ^*TOTAL*^ ratio. Conversely, *C9orf72* ^*RE*^ and *SOD1* I114T patients did not exhibit any notable changes in the relative expression of the Ca^2+^‐permeable subunits *GluA1*, *GluA3* and GluA4 in the prefrontal cortex. The data therefore demonstrate that the observed dysregulation of AMPAR subunit expression in the prefrontal cortex is specific to sALS patients. Furthermore, the fact that the prefrontal and motor cortex of sALS and *C9orf72* ^*RE*^ patients shows overlapping TDP‐43 pathology (Table 1) suggests that TDP‐43 cannot be the sole pathogenic substrate that generates altered Ca^2+^‐permeable AMPAR subunit expression in the cortical regions of sALS patients.

## Discussion

This study has provided the first comparative study of AMPAR subunit expression across post‐mortem brain regions (spinal cord – anterior horn, motor cortex – BA4 and prefrontal cortex – BA9) of the most prevalent genetic ALS patient cohorts; sporadic ALS, *SOD1* mutation and *C9orf72* ^*RE*^. To do this we employed a high resolution *in situ* hybridisation technique, BaseScope, which permitted a quantitative assessment of AMPAR subunit expression at the single‐transcript and single‐cell level previously not detailed in control individuals or ALS patients. Whilst it is not possible in post‐mortem tissue, using laser capture dissection or *in situ* hybridisation, to capture a neuron in its entirety, we have sampled multiple cells in multiple regions to try to limit sampling bias, as other studies have done 9. Using these methods, our data indicate extensive AMPAR subunit dysregulation in all brain regions examined from sALS patients. However, AMPAR dysregulation was observed to be restricted to spinal motor neurons for *SOD1* I114T and *C9orf72* ^*RE*^ patients.

AMPAR subunit dysregulation in spinal motor neurons has been described in several ALS models 20 and in human ALS patients 9, where mechanistic disease hypotheses have centred on an increased vulnerability to excitotoxicity due to an increased Ca^2+^‐permeable AMPAR population in these cells. Previously 15, we showed that *C9orf72* ^*RE*^ spinal motor neurons exhibited an increased functional Ca^2+^‐permeable AMPAR population underpinned by an increase in the abnormal expression of the *GluA1* subunit and, in this study, is reflected as a reduction in the *GluA2/GluA* ^*TOTAL*^ expression ratio. Noting the expression of *GluA1* in adult spinal motor neurons is consistently reported to be absent or negligible 15, we found that sALS patient spinal motor neurons exhibit equivalent substantial dysregulation of *GluA1* transcripts and protein. This indicates that sALS and *C9orf72* ^*RE*^ spinal motor neurons, which both exhibit TDP‐43 pathology, may share pathological mechanisms. Indeed, the *GluA2/GluA* ^*TOTAL*^ expression ratio for sALS spinal motor neurons was, surprisingly, not different to that of control individuals and the observed reduced expression of Ca^2+^‐permeable GluA4 subunit may instead reflect compensatory mechanisms for the increased *GluA1* expression in sALS patients. These specific data suggest to us that sALS patients do not exhibit differences in the relative level of the Ca^2+^‐permeable AMPAR population in spinal motor neurons. However, a functional Ca^2+^‐permeable AMPAR population still cannot be ruled out given that AMPAR assembly, trafficking and localisation is highly subunit dependent 21 and that the underlying profile of AMPAR subunit expression is different in sALS patients to that of control individuals.

*GluA1* was not expressed by *SOD1* I114T patients, which do not exhibit TDP‐43 pathology, confirming *GluA1* dysregulation is associated with common TDP‐43 pathology. However, we observed that only Ca^2+^‐permeable AMPARs could be expressed by *SOD1* I114T spinal motor neurons since *GluA2* was found not to also be expressed. These data are the first to be presented in the context of human ALS patients with *SOD1* mutations, though are in broad agreement with previous studies of mutant *Sod1* rodent models of ALS that implicate a reduction in *GluA2* expression in spinal motor neurons 11, 13, 23, 24, 25, 26, 27. Despite mechanistic divergence at the AMPAR subunit transcriptional level, it appears that *SOD1* mutant and *C9orf72* ^*RE*^ patient spinal motor neurons show a functional convergence through an increase in the relative Ca^2+^‐permeable AMPAR population.

Inefficient (glutamine to arginine) RNA editing of the GluA2 subunit also generates an increase in Ca^2+^‐permeable AMPARs and has been evidenced in sALS post‐mortem spinal motor neurons 10, but not *SOD1* mutation post‐mortem motor neurons 21, *C9orf72* ^*RE*^ motor neurons 15 and whole spinal cord lysates from *C9orf72* ^*RE*^ patients 27. We examined *GluA2* editing in sALS patient‐derived motor neurons and found the editing of *GluA2* to be highly efficient.

ALS is also typified by the degeneration of upper motor neurons and several other brain regions are also affected by TDP‐43 deposition. We therefore performed a comparative quantitative assessment of AMPAR subunit expression in the ALS motor cortex (BA4) and pre‐frontal cortex (BA9). Our data indicated that *SOD1* I114T and *C9orf72* ^*RE*^ patients did not exhibit any change in *GluA2/GluA* ^*TOTAL*^ expression or subunit profiles with respect to control individuals in the cortical areas investigated and indicate that AMPAR dysregulation is restricted to spinal motor neurons. Despite sharing equivalent TDP‐43 pathology with *C9orf72* ^*RE*^ patients, dysregulation of AMPAR subunit expression in the motor and prefrontal cortex of sALS patients is, however, prominent. The *GluA2/GluA* ^*TOTAL*^ expression ratio in the pre‐frontal cortex and motor cortex in sALS is lower due to an increase in *GluA1* expression and a reduction in *GluA2* expression, respectively. TDP‐43 pathology alone therefore cannot account for the observed differential spatial AMPAR dysregulation. These scenarios lead to an increase in Ca^2+^‐permeable AMPARs in sALS motor and pre‐frontal cortex and suggest that they may contribute to excitotoxic‐mediated cell death. In this regard, we highlight that *GluA2/GluA* ^*TOTAL*^ expression ratios are estimations and not direct measurements of the functional Ca^2+^‐permeable AMPAR composition.

Our data indicate that neuronal AMPAR subunit expression is heterogeneously regulated between ALS cases with different genetic backgrounds and between different brain regions within each case type. Furthermore, given that sALS patients exhibited altered AMPAR subunit regulation in all regions examined and *C9orf72* ^*RE*^ patients only in anterior horn motor neurons, it appears that classical TDP‐43 pathology can only be associated with aspects of anterior horn motor neuron, but not cortical, AMPAR subunit dysregulation. Nonetheless, with the potential exception of sALS anterior horn motor neurons, all observed perturbed AMPAR subunit expression is consistent with a convergence on an increased relative Ca^2+^‐permeable AMPAR population, which is thought to underpin an increased vulnerability to glutamate‐mediated neurotoxicity.

## Author contributions statement

JMG, MRL, SC and CS designed the study. JMG, MRL, KM, BTS, SB, SC and CS prepared the samples and carried out experiments. JMG, MRL, SC and CS conceived experiments and analysed data. JMG, MRL, BTS, SC and CS interpreted the data. JMG, MRL, SC and CS wrote the manuscript. All authors had final approval of the submitted and published versions. JMG and MRL contributed to the study equally.

## References

[1] Renton AE , Majounie E , Waite A , *et al* A hexanucleotide repeat expansion in C9ORF72 is the cause of chromosome 9p21‐linked ALS‐FTD. Neuron 2011; 72: 257–268. 21944779 10.1016/j.neuron.2011.09.010 PMC3200438

[2] Black HA , Leighton DJ , Cleary EM , *et al* Genetic epidemiology of motor neuron disease‐associated variants in the Scottish population. Neurobiol Aging 2017; 51: 178.e11–178.e20. 10.1016/j.neurobiolaging.2016.12.013 PMC5302213 28089114

[3] Scotter EL , Chen HJ , Shaw CE . TDP‐43 proteinopathy and ALS: insights into disease mechanisms and therapeutic targets. Neurotherapeutics 2015; 12: 352–363. 25652699 10.1007/s13311-015-0338-x PMC4404432

[4] Taylor JP , Brown RH Jr , Cleveland DW , *et al* From genes to mechanism. Nature 2016; 539: 197–206. 27830784 10.1038/nature20413 PMC5585017

[5] Balendra R , Isaacs AM . C9orf72‐mediated ALS and FTD: multiple pathways to disease. Nat Rev Neurol 2018; 14: 544–558. 30120348 10.1038/s41582-018-0047-2 PMC6417666

[6] Cleveland DW , Rothstein JD . From Charcot to Lou Gehrig: deciphering selective motor neuron death in ALS. Nat Rev Neurosci 2001; 2: 806–819. 11715057 10.1038/35097565

[7] Traynelis SF , Wollmuth LP , McBain CJ , *et al* Glutamate receptor ion channels: structure, regulation, and function. Pharmacol Rev 2010; 62: 405–496. 20716669 10.1124/pr.109.002451 PMC2964903

[8] Williams TL , Day NC , Ince PG , *et al* Calcium‐permeable alpha‐amino‐3‐hydroxy‐5‐methyl‐4‐isoxazole propionic acid receptors: a molecular determinant of selective vulnerability in amyotrophic lateral sclerosis. Ann Neurol 1997; 42: 200–207. 9266730 10.1002/ana.410420211

[9] Kawahara Y , Kwak S , Sun H , *et al* Human spinal motoneurons express low relative abundance of GluR2 mRNA: an implication for excitotoxicity in ALS. J Neurochem 2003; 85: 680–689. 12694394 10.1046/j.1471-4159.2003.01703.x

[10] Kawahara Y , Ito K , Sun H , *et al* Glutamate receptors: RNA editing and death of motor neurons. Nature 2004; 427: 801. 14985749 10.1038/427801a

[11] Van Damme P , Braeken D , Callewaert G , *et al* GluR2 deficiency accelerates motor neuron degeneration in a mouse model of amyotrophic lateral sclerosis. J Neuropathol Exp Neurol 2005; 64: 605–612. 16042312 10.1097/01.jnen.0000171647.09589.07

[12] Van Den Bosch L , Van Damme P , Bogaert E , *et al* The role of excitotoxicity in the pathogenesis of amyotrophic lateral sclerosis. Biochim Biophys Acta 2006; 1762: 1068–1082. 16806844 10.1016/j.bbadis.2006.05.002

[13] Van Damme P , Bogaert E , Dewil M , *et al* Astrocytes regulate GluR2 expression in motor neurons and their vulnerability to excitotoxicity. Proc Natl Acad Sci U S A 2007; 104: 14825–14830. 17804792 10.1073/pnas.0705046104 PMC1976195

[14] Shi Y , Lin S , Staats KA , *et al* Haploinsufficiency leads to neurodegeneration in C9ORF72 ALS/FTD human induced motor neurons. Nat Med 2018; 24: 313–325. 29400714 10.1038/nm.4490 PMC6112156

[15] Selvaraj BT , Livesey MR , Zhao C , *et al* *C9ORF72* repeat expansion causes vulnerability of motor neurons to Ca^2+^‐permeable AMPA receptor‐mediated excitotoxicity. Nat Commun 2018; 9: 347. 29367641 10.1038/s41467-017-02729-0 PMC5783946

[16] Mackenzie IR , Bigio EH , Ince PG , *et al* Pathological TDP‐43 distinguishes sporadic amyotrophic lateral sclerosis from amyotrophic lateral sclerosis with SOD1 mutations. Ann Neurol 2007; 61: 427–434. 17469116 10.1002/ana.21147

[17] Tölle TR , Berthele A , Zieglgänsberger W , *et al* The differential expression of 16 NMDA and non‐NMDA receptor subunits in the rat spinal cord and in periaqueductal gray. J Neurosci 1993; 13: 5009–5028. 8254358 10.1523/JNEUROSCI.13-12-05009.1993 PMC6576409

[18] Rekling JC , Funk GD , Bayliss DA , *et al* Synaptic control of motoneuronal excitability. Physiol Rev 2000; 80: 767–852. 10747207 10.1152/physrev.2000.80.2.767 PMC4764886

[19] Mackenzie IR , Frick P , Neumann M . The neuropathology associated with repeat expansions in the C9ORF72 gene. Acta Neuropathol 2014; 127: 347–357. 24356984 10.1007/s00401-013-1232-4

[20] Kwak S , Hideyama T , Yamashita T , *et al* AMPA receptor‐mediated neuronal death in sporadic ALS. Neuropathology 2010; 30: 182–188. 20102521 10.1111/j.1440-1789.2009.01090.x

[21] Pick JE , Ziff EB . Regulation of AMPA receptor trafficking and exit from the endoplasmic reticulum. Mol Cell Neurosci 2018; 91: 3–9. 29545119 10.1016/j.mcn.2018.03.004 PMC6128777

[22] Pieri M , Gaetti C , Spalloni A , *et al* Alpha‐Amino‐3‐hydroxy‐5‐methyl‐isoxazole‐4‐propionate receptors in spinal cord motor neurons are altered in transgenic mice overexpressing human Cu, Zn superoxide dismutase (Gly93→Ala) mutation. Neuroscience 2003; 122: 47–58. 14596848 10.1016/j.neuroscience.2003.07.003

[23] Tateno M , Sadakata H , Tanaka M , *et al* Calcium‐permeable AMPA receptors promote misfolding of mutant SOD1 protein and development of amyotrophic lateral sclerosis in a transgenic mouse model. Hum Mol Genet 2004; 13: 2183–2196. 15294873 10.1093/hmg/ddh246

[24] Kuner R , Groom AJ , Bresink I , *et al* Late‐onset motoneuron disease caused by a functionally modified AMPA receptor subunit. Proc Natl Acad Sci U S A 2005; 102: 5826–5831. 15827116 10.1073/pnas.0501316102 PMC556301

[25] Petri S , Krampfl K , Hashemi F , *et al* The mRNA expression of AMPA type glutamate receptors in the primary motor cortex of patients with amyotrophic lateral sclerosis: an in situ hybridization study. Neurosci Lett 2004; 360: 170–174. 15082160 10.1016/j.neulet.2004.03.002

[26] Camerino GM , Fonzino A , Conte E , *et al* Elucidating the contribution of skeletal muscle ion channels to amyotrophic lateral sclerosis in search of new therapeutic options. Sci Rep 2019; 9: 3185. 30816241 10.1038/s41598-019-39676-3 PMC6395744

[27] Moore S , Alsop E , Lorenzini I , *et al* ADAR2 mislocalization and widespread RNA editing aberrations in C9orf72‐mediated ALS/FTD. Acta Neuropathol 2019; 138: 49. 30945056 10.1007/s00401-019-01999-w PMC6750285

